# Both Soil Bacteria and Soil Chemical Property Affected the Micropredator Myxobacterial Community: Evidence from Natural Forest Soil and Greenhouse Rhizosphere Soil

**DOI:** 10.3390/microorganisms8091387

**Published:** 2020-09-10

**Authors:** Yang Zhou, Xianjiao Zhang, Qing Yao, Honghui Zhu

**Affiliations:** 1 Guangdong Provincial Key Laboratory of Microbial Culture Collection and Application, Guangdong Open Laboratory of Applied Microbiology, State Key Laboratory of Applied Microbiology Southern China, Guangdong Microbial Culture Collection Center (GDMCC), Guangdong Institute of Microbiology, Guangdong Academy of Sciences, Guangzhou 510070, China; zhouyang@gdim.cn (Y.Z.); zhangxj@gdim.cn (X.Z.); 2 College of Horticulture, South China Agricultural University, Guangdong Province Key Laboratory of Microbial Signals and Disease Control, Guangdong Engineering Research Center for Litchi, Guangdong Engineering Research Center for Grass Science, Guangzhou 510642, China

**Keywords:** forest soil myxobacteria, soil bacterial community, soil bacterial abundance, soil chemical property

## Abstract

Myxobacteria are abundant micropredators in soil, and are social bacteria with multicellular behavior and producers of versatile secondary metabolites. The interaction between predator and prey populations is an important component in the soil microbial food web, and this is expected to shape the composition and dynamics of microbial communities. Here we hypothesize the regulation of bacterial abundance and community composition on soil myxobacterial community. Field investigation indicated that the relative abundance of Myxococcales in subtropical and tropical forest soil from South China was 1.49−4.74% of all the 16S rRNA gene sequences, and myxobacterial community composition differed between subtropical and tropical forest. The canonical correspondence analysis and variation partitioning analysis indicated that biotic factor (bacterial community composition) showed slightly stronger explanation for variation of myxobacteria than soil properties (soil pH and soil organic matter). Based on the rhizosphere bacterial network, the greenhouse mesocosm experiment showed that most of the myxobacterial links were with Gram-negative bacteria, except that some nodes from Haliangiacea and Polyangiaceae interacted with actinomycetes and actinomycetes-like Gram-positive bacteria. We inferred that myxobacteria preferential predation on specific bacterial taxa may explain the influence of bacteria on myxobacterial community. Further study confirming the biological process of myxobacterial predation in situ is necessary to advance the understanding of the ecological role of predation behavior in the microbial world.

## 1. Introduction

Myxobacteria are known as micropredators, social bacteria with multicellular behavior and producers of versatile secondary metabolites. The myxobacterial group of vegetative cells secrete antibiotics, hydrolases and other bacteriolytic compounds to kill and lyse the prey bacteria [1,2]. Myxobacteria are reported to be highly adaptable cosmopolitans distributed in various environments despite some locally presented taxa [3,4]. Terrestrial ecosystems are typical habitats for myxobacteria [3,4,5]. Recent culture-independent studies have demonstrated an astonishingly high diversity of uncultured myxobacteria [4,6,7,8]. However, details about the composition and taxonomy of myxobacterial community are poorly understood.

Previous studies revealed that the composition and diversity of soil bacterial communities are shaped by soil pH, soil organic matter (SOM), and other environmental characteristics at global and regional scales [9,10,11]. However, only a few studies have reported the details of diversity and distribution for specific bacteria [12,13,14,15]. As one of the most abundant bacteria in soil, the ecological distribution of myxobacteria might be associated with environmental factors [3,16]. The phylogenetic separation of soil- and marine-originated myxobacteria indicates environmental selection [6]. Furthermore, mean annual temperature, soil pH, carbon and nitrogen ratio, and organic carbon content have been reported to be strongly correlated with myxobacterial abundance [16]. Notably, myxobacteria cells can aggregate to form fruiting bodies filled with resistant myxospores under starvation [3,17], which is a favorable strategy for their surviving in different and changing environments. Therefore, we can expect that the ability of forming resistant myxospores contributes to their wide adaptability, which may weaken the influence of environmental factors on myxobacteria. Moreover, the dependence on prey as nutrient source weakens the association of myxobacteria with ambient environment.

The interaction between myxobacteria and prey population is an important component of microbial food web [18,19]. Previous axenic co-culture experiments have shown variant efficiencies of myxobacterial predation against different prey species [20]. While *Myxococcus xanthus* has been reported to exhibit predatory activity against a broad range of prey, some prey bacteria characteristics, such as bacillaene production, sporulation [21] and exopolysaccharide galactoglucan formation [22], can prevent or alleviate myxobacterial predation. Therefore, it is reasonable to speculate that the species of bacteria (prey or non-prey) may influence the assembly of myxobacteria community, because the prey can support its predators, but the non-prey cannot. Chen et al. [23] evidenced the regulation of prey bacteria on the diversity of predator *Bdellovibrio*, a phylogenetically relevant predatory bacteria group to myxobacteria within Deltaproteobacteria. However, how the prey bacteria regulate soil predatory myxobacteria at the community level is far from understood.

While most studies report the abiotic factors in shaping microbial community, studies focusing on the influence of microbial interactions on microbial community and ecological function are much fewer in number [24,25,26]. As facultative predators, soil myxobacteria can use macromolecules (such as organic matter, dead cells) and prey on live microorganisms as food sources. Therefore, both abiotic and biotic factors are expected to be important factors shaping the myxobacterial community. We hypothesized that (1) soil myxobacterial community would be influenced by soil bacteria; (2) the relative contributions of measured soil bacterial and abiotic factors (e.g., soil pH and SOM) might differ in affecting the myxobacterial community. In this study, both field investigation and a greenhouse mesocosm experiment were conducted to explore the effect of soil bacterial factors (abundance and community composition) on the predatory myxobacteria community. Firstly, we collected soil samples from subtropical and tropical forests, and soil myxobacterial communities were explored based on 16S rRNA gene sequencing; then bacterial community data from rhizosphere soil in the greenhouse mesocosm experiment were included to illustrate the interactions between myxobacteria and other bacteria as well as soil parameters under the enrichment of soil bacteria with plant presence.

## 2. Materials and Methods

### 2.1. Study Sites and Field Sample Collection

The forests in South China are in a broad area from subtropical to tropical zones, which is a treasure trove of terrestrial diversity. In particular, the subtropical forests in South China include the rarely moist biosphere near the Tropic of Cancer [27]. Previous studies have reported the high diversity of myxobacteria in different forests by a culture-dependent method [3]. Therefore, we collected soil samples of subtropical and tropical forests in South China to study the myxobacterial community. The sampling sites included one subtropical forest in Guangdong Province (Chebaling National Nature Reserve, E 114°08′19″, N 24°42′30″, CB) and three tropical forests ranging from east to west of Hainan Island (Diaoluoshan Natural Nature Reserve, E 109°46′23″, N 18°25′49″, DL; ES; Limushan Natural Nature Reserve, E 109°46′05″, N 19°12′20″, LM; Esongling mountain, E 109°26′08″, N 19°11′56″). CB is located in the transition zone from the southern subtropical area to the middle subtropical area and is dominated by primary evergreen broad-leaved forest with moist, moderate subtropical monsoon climate. The average temperature is 19.6 °C, and the annual precipitation is 1467 mm. The sampling sites of CB located at the mountain areas along the Shixing and Quannan county, which is tectonically a part of the fold system in South China with light metamorphic sand shale and acid volcanic rock. The soil type is typical red soil [28]. DL has a tropical monsoon climate with a mean monthly temperature range from 15 °C in January to 28 °C in July. Annual precipitation is 1800 to 2000 mm. Our sampling sites were mainly located in Lingshui county. The geology is mainly granite and the soil type is acid red soil [29]. LM mountains lies in the middle of Hainan Island with the tropical monsoon climate. The annual temperature is 22.5 °C and the annual precipitation is 2343 mm. The sampling sites were the area of pristine tropical rainforest located Qiongzhong county. The soil types are laterite and latosol [30]. ES is located in Baisha county with tropical oceanic monsoon climate. The annual temperature is 22.6 °C and the annual precipitation is 1896 mm. The soil types belong to latosol and dry red soil [31].

At each site, 10−30 plots (each approximately 10 m × 10 m) were established depending on the terrain with more than 20 m between adjacent plots. Soil samples were collected from the top 10 cm. Three soil cores were collected at each plot, and these soil samples were sieved through a 2 mm mesh and pooled into a composite for thorough homogenization. All soil samples were transported to the laboratory on ice. Samples were divided into two aliquots in the laboratory, with one aliquot stored at −80 °C for soil DNA extraction and the other air-dried for the measurement of soil parameters.

### 2.2. Measurement of Soil pH and Organic Matter Content

Soil property was reported to be correlated with soil microbial community [9,10,11]. Here we also associated soil myxobacteria with soil property. According to soil parameter data from general survey (http://www.geodata.cn), other potential parameters affecting microbial community (such as soil nitrogen, phosphorus, potassium) showed a small variance among our sampling sites, which may exert less important influence than pH and SOM. Therefore, we measured the soil pH and SOM to associate with myxobacteria. As the potential nutrient sources of myxobacteria, SOM was analyzed using the dichromate oxidation-titration method [32]. Because soil pH was reported to be an important soil factor affecting the biogeography of microbial community [10], we also measured soil pH using a glass electrode (Sartorius PB-10) after the suspension of 10 g soil in 25 mL distilled water.

### 2.3. Soil DNA Extraction and HiSeq Sequencing

Soil total DNA was extracted using PowerSoil DNA Isolation Kit (MoBio Laboratories Inc., Carlsbad, CA, USA) according to the manufacturer’s protocol. The 338F/806R primer sets were used to amplify V3+V4 region of bacterial 16S rRNA gene, with the reserve primer fused with barcode sequences for each sample [33]. PCR reaction contained 30 ng template DNA, 0.2 μmol^−1^ of forward and reserved primers, and high-fidelity PCR mix (New England Biolabs, Ipswich, MA, USA). The PCR reaction were conducted as described previously [34]. Then the retrieved PCR products were used for sequencing library construction with the NEB Next Ultra DNA Library Prep Kit for Illumina following manufacturer’s recommendations. The sequencing was conducted on an Illumina MiSeq platform. The sequence data have been deposited in the NCBI Sequence Read Archive (SRA) with the accession number PRJNA526754.

### 2.4. Bioinformatic and Statistical Analysis

Raw reads were de-multiplexed, quality-filtered and analyzed using the open source bioinformatic tool QIIME2 version 2018.6 [35]. Low-quality bases were first removed from the reads and amplicon sequence variants (ASV) were generated using the Deblur algorithm. Deblur aligned the sequences together into ASV based on an upper error rate bound along with a constant probability of indels and the mean read error rate, and the predicted error-derived sequences from neighboring sequences were removed [36]. The annotation was performed against the database Sliva-132-99-nb classifier [37].

The sequences annotated to Myxococcales were used to generate the myxobacterial community data for all samples. Nonmetric multidimensional scaling (NMDS) analysis was conducted to visualize the myxobacterial population among samples in PAST software with the pairwise Bray-Curtis distance matrix (Paleontological Statistics version 3.11) [38]. Analysis of similarity (ANOSIM) was performed in PAST to test the differences of samples with myxobacterial community by pairwise Bray–Curtis distance.

The difference of soil myxobacteria relative abundance and alpha diversity among sampling sites were analyzed with ANOVA and Tukey’s multiple range test in R (version 3.4.0; R development core team, 2017), respectively. The relationship between soil bacteria and myxobacterial abundance and community composition were revealed by Spearman correlation and Mantel test in R. Canonical correspondence analysis (CCA) was used to identify soil bacterial community and chemical property (soil pH and SOM) affecting soil myxobacterial community composition. We used the first and second principal coordinates analysis (PCoA) axes to represent bacterial community composition in the CCA, according to Zhang et al. [39]. The first and second axes show near 60% variations of bacterial communities of all the forest soil samples (Table S1), which represents a relatively high proportion of soil bacterial community variations. The PCoA, CCA and Monte Carlo permutation tests were conducted with the “vegan” package [40] in R statistical software. To resolve the explanatory power of different factors (bacterial community and soil parameters) in relation to the soil myxobacterial community, we conducted variation partitioning analysis (VPA) in R using the “varpart” function in the “vegan” package [40].

### 2.5. Myxobacterial Sequence Collection and Data Analysis from the Mesocosm Experiment under Greenhouse Conditions

We collected bacterial community data from a greenhouse mesocosm experiment with enriched bacteria from rhizosphere by planting, exploring how the bacteria abundance and the biotic/abiotic interactions affect myxobacterial community. The greenhouse experiment design and procedures have been reported previously [34]. Briefly, six plant species [*Stylosanthes guianensis* (Aubl.) Sw., *Trifolium pratense* L., *Medicago sativa* L., *Paspalum natatum* Flüggé, *Festuca arundinacea* L. and *Lolium perenne* L.] were planted in pots with soil collected from a subtropical forest (E 112°54′19″, N 22°40′20″). The rhizosphere soil and control samples without planting were collected after plant harvest. Then soil pH, SOM content and other properties (dissolved organic carbon, total and available nitrogen, phosphate, potassium) were measured. Soil total DNA was extracted for the bacterial 16S rRNA gene copy numbers quantification and high-throughput sequencing (NCBI sequence read archive accession number: SRX2589894).

Myxobacterial community data in the greenhouse experiment were obtained as described above in the forest investigation. The effect of plant presence on soil myxobacterial abundance was analyzed by t-test. The correlations of bacteria abundance, myxobacteria and soil parameters were indicated by Spearman rank correlation in R. The bacterial factors included bacterial absolute abundance and community composition (PCoA axes was used to present bacterial community, Bact1 and Bact2 indicated in Table S2 as described above for the forest field investigation). Bio-env and RDA were used to identify abiotic and bacterial factors affecting the myxobacterial population with “vegan” package [40].

We used rhizosphere bacterial community composition data and soil parameters to construct a powerful network for interaction analysis according to previous studies [41,42]. The network analysis was performed with a random matrix theory (RMT) based online pipeline (http://ieg4.rccc.ou.edu/MENA) according to Deng et al. [43], including four steps: (1) ASVs detected in half or more than half of the total samples with relative abundance > 0.01% were kept for network construction; (2) the default cut-off (0.8) for the similarity matrix was adopted to obtain an adjacency matrix; (3) calculations of network properties and random network properties; and (4) network visualization in the Cytoscape software [44] and Gephi platform [45].

## 3. Results

### 3.1. Myxobacterial Abundance and Community Composition in Subtropical and Tropical Forest Soil

**Figure 1:**
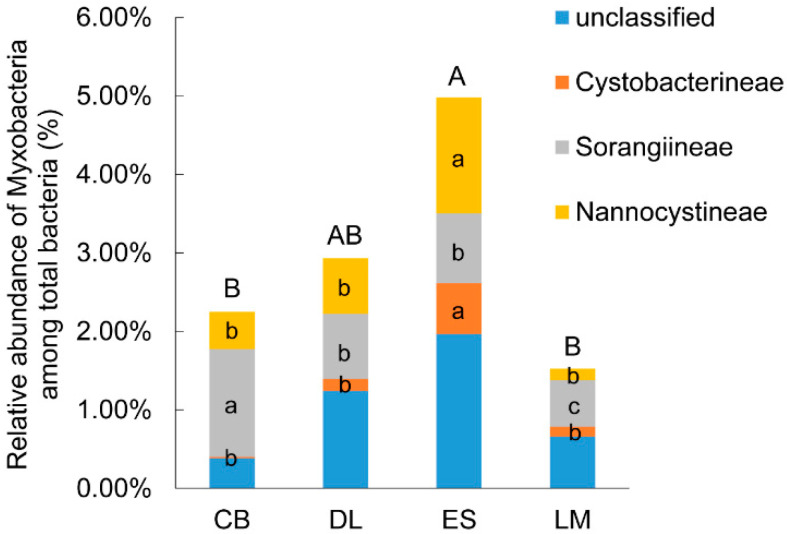
The relative abundance and community composition at the suborder level of soil myxobacteria among total bacteria from subtropical and tropical forest soil. The different uppercase letters above each column indicate significant difference of total myxobacteria abundance among sampling sites, and the different lowercase letters embedding the center of each stacking indicate significant difference of each suborder abundance among sites (*p* < 0.05, Tukey’s multiple range test). CB, Chebaling (subtropical forest soil); DL, Diaoluoshan (tropical forest soil); ES, Esongling (tropical forest soil); LM, Limushan (tropical forest soil).

The relative abundance of Myxococcales in subtropical and tropical forest soil from South China ranged from 1.49% to 4.74% (Figure 1), which ranked 11th most abundant order among all bacteria. ES soil from tropical forest had a significantly higher abundance of myxobacteria than that of CB and LM. While CB showed the highest abundance of Sorangiineae and the lowest abundance of Cystobacterineae, ES displayed the highest abundance of Cystobacterineae and Nannocystineae (Figure 1). DL showed moderate abundances of the three suborders. Taxonomy annotation indicated that most of the sequences were from uncultured Myxococcales. More than 50% of sequences were from the three known suborders of myxobacteria, with Cystobacterineae showing the lowest abundance. As with previous studies, myxobacteria can be divided into predators and cellulose degraders according to the specialization in degradation biological macromolecules with the former accounting for the majority [2]. It is worth noting that we did not detect sequences from cellulose-degraders (*Sorangium* and *Byssovorax*), that is, all the sequences in this study were from predatory myxobacteria.

**Figure 2:**
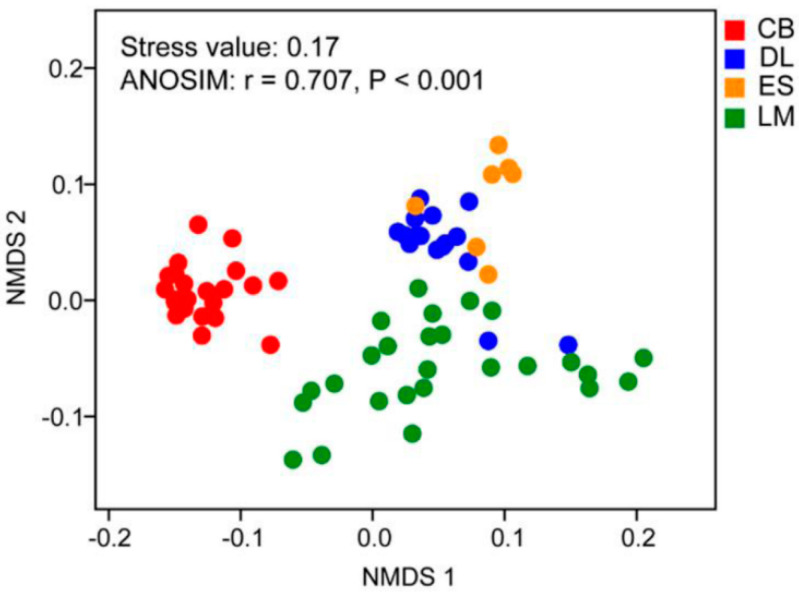
NMDS of Bray–Curtis similarity matrix of soil myxobacterial community from subtropical and tropical forest soil based on amplicon sequence variants (ASV) abundance. Stress value and analysis of similarity are shown in the upper left of the graph. CB, Chebaling (subtropical forest soil); DL, Diaoluoshan (tropical forest soil); ES, Esongling (tropical forest soil); LM, Limushan (tropical forest soil).

Sampling site affected soil myxobacterial community composition (ANOSIM: R = 0.707, *p* < 0.001), and the samples from the subtropical forest were distinguished from the tropical forest samples as demonstrated in the NMDS plot (Figure 2). However, the myxobacterial communities from tropical forests were difficult to separate well from each other by sampling sites (Figure 2).

### 3.2. Forest Soil Bacteria as a Biotic Factor Correlated with Myxobacteria

Spearman rank correlation analysis indicated that the abundance of some bacterial groups at phylum level was significantly correlated with myxobacterial abundance, such as Acidobacteria, Bacteroidetes, Chloroflexi, Firmicutes and Gemmatimonadetes (Table 1). The abundance of the main group (such as Proteobacteria, Acidobacteria and Actinobacteria) and some other soil groups (Bacteroidetes, Chloroflexi, Firmicutes and Gemmatimonadetes) was significantly correlated with the myxobacterial community composition (Table 1).

### 3.3. The Contribution of Biotic and Abiotic Factors in Relation to Forest Myxobacterial Community

**Figure 3:**
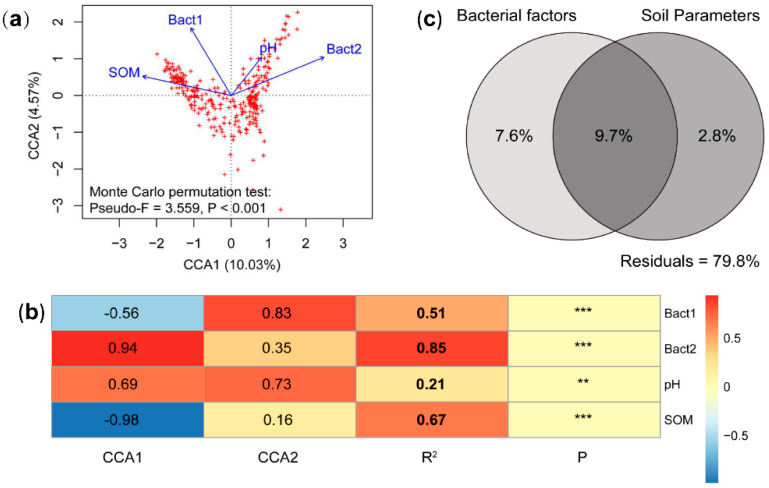
The contribution of soil abiotic and bacterial factors affecting myxobacterial community of subtropical and tropical forest soil. (**a**) CCA of soil myxobacterial community composition constrained by soil bacterial community (Bact1, Bact2) and soil abiotic parameters (pH, SOM); Bact1 and Bact2 indicate first and second principal components generated from PCoA using soil microbial community composition data (the first and second principal components explained 57% of the variation of the 71 soil bacterial communities). (**b**) Correlations between ordination axis and constrained factors and their significance indicated by Monte Carlo permutation test; R^2^ means the effect size of specific factor on soil myxobacteria, and *p* < 0.05 indicates a significant effect on myxobacterial community. (**c**), VPA indicated the effects of soil abiotic and bacterial factors on the myxobacterial community composition; the data present the percentage of variations explained by the factors.

Many studies have analyzed abiotic factors affecting soil microbial community. Here we included both biotic factors (soil bacteria) and abiotic factors (pH and SOM) to explore their influences on myxobacterial community. The CCA indicated a significant model in explaining the myxobacterial community variation with the measured soil chemical parameters (pH and SOM) and bacteria community as constrained factors (Figure 3a). Based on correlation analysis, soil bacterial community (the second axis of PCoA from soil bacterial community and the first and second PCoA axis indicated 30.91% and 26.13% variation of bacterial community, respectively; Table S1) were the strongest factors affecting myxobacterial community among all the adopted factors (effect size R^2^ shown in Figure 3b).

The VPA showed that bacterial community composition and the measured soil parameters explained more than 20% of variation of the myxobacterial community from forest soil in South China. The bacterial factor explained 17.3% of variation of myxobacterial community composition, and soil parameters (pH and SOM) accounted for 12.5% (Figure 3c). In addition, these two factors showed strong interaction in explaining the myxobacterial variation, which may reflect the obvious associations with soil bacteria and soil pH and SOM (Figure 3c).

### 3.4. The Influence of Bacterial Abundance and Interactions on Myxobacteria in the Greenhouse Mesocosm Experiment

We indicated that soil bacterial community composition and the abundance of specific bacterial phyla were significantly correlated with myxobacterial abundance and community in subtropical and tropical forest soil from South China. Furthermore, we collected the myxobacterial sequences and abundance data from rhizosphere soil bacterial 16S rRNA gene sequencing data in a previous reported experiment [34]. The enriched bacteria cells and slightly different microbiomes were obtained by planting grasses in the controlled mesocosm experiment, which was used to explore the interactions among myxobacteria, bacteria and soil parameters under controlled conditions in this study. In addition, we can use this controlled system to confirm the results from natural forests.

**Figure 4:**
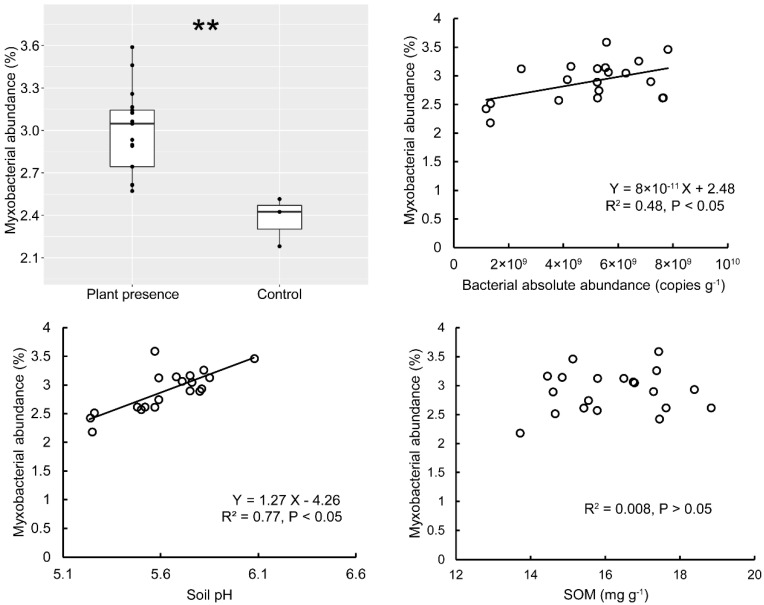
The relative abundance of soil myxobacteria and the correlation with soil bacteria abundance, soil pH and SOM content from the greenhouse mesocosms experiment. The asterisk (**) indicates significant difference between plant presence and the no-plant control. Linear functions, coefficients and *p* values are used to describe the significant correlations.

As expected, an approximately 5-fold increase of soil bacterial abundance was detected with plant presence [34]. The plant presence significantly increased the relative abundance of Myxococcales (T-test, *p* = 0.003, Figure 4). The bacterial abundance was positively correlated with Myxococcales abundance (R^2^ = 0.48, *p* < 0.05). Soil pH was also correlated with myxobacterial abundance (R^2^ = 0.77, *p* < 0.05), but SOM was not (Figure 4). Soil bacterial community composition and abundance were closely associated with the myxobacterial community, while abiotic soil parameters, e.g., pH and SOM (other measured soil variables were excluded by Bio-env in R) were minor factors (Figure S1, Table S3).

**Figure 5:**
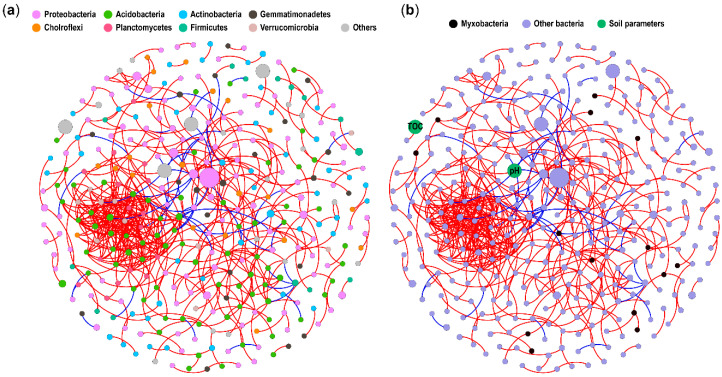
The co-occurrence networks showing the associations of soil myxobacteria, other bacteria and soil parameters (pH, SOM). (**a**) The nodes coded with different colors represent individual ASV and soil parameters. (**b**) The nodes coded with different colors represent myxobacteria, other bacteria and soil parameters. The edges connecting nodes correspond to statistically significant correlations between nodes and the edges in red and blue indicate positive interactions and negative interactions, respectively. Node size is the mean abundance of each ASV except the node representing soil parameter with constant size to distinguish from bacterial nodes.

Network analysis of bacterial co-occurrences, as measured by abundance correlations of ASVs, can help decipher microbial association [41]. In addition, network can also link microbial taxa to environmental parameters [41,42]. With the rhizosphere bacterial community data and soil parameters, we performed the co-occurrence network analysis to explore the links among myxobacteria, bacteria and soil parameters affecting soil myxobacteria. The rhizosphere soil bacteria network was confirmed to be non-random (Table S4). The 35% of myxobacterial ASVs interacted with other bacteria in the network under a cutoff value of 0.8, and most of the myxobcterial nodes seemed to interact with Gram-negative bacteria (Figure 5). However, direct links between soil parameters and myxobacteria were not detected within the network (Figure 5).

## 4. Discussion

The biogeography of the microbial community indicated the effect of environmentally abiotic factors on the distribution of microorganisms [9,10,11]. Moreover, microorganisms are also engaged in complex interactions with plants, animals, and other microbes, which can have beneficial, neutral, or harmful influences on members of the community [26,34,46,47]. In this study, we investigated the myxobacterial community in subtropical and tropical soil, a widely distributed micropredator, and the results demonstrated that soil bacterial abundance and composition were associated with myxobacterial community. We also quantified the relative importance of measured soil abiotic parameters and bacterial factors in relation to the soil myxobacterial communities and found a slightly stronger explanation of bacterial factors than soil pH and SOM.

### 4.1. Myxobacteria Relative Abundance and Community Composition in Subtropical and Tropical Forest Soil from South China

Global scale distribution based on the culture-dependent method indicates that tropical areas are typical environments for myxobacteria [3]. However, the knowledge of myxobacterial distribution has mostly been retrieved on the basis of strain isolation. Via pyrosequencing of a single soil sample, Zhou et al. [16] found that the relative abundance of myxobacteria was 4.1% of the whole bacterial community. We detected that sequences of Myxococcales accounted for 1.49−4.74% in 16S rRNA gene libraries from subtropical and tropical forest soil in South China. Combining the relative abundance of myxobacteria and the total bacterial 16S rRNA gene copy numbers, we estimated that the absolute abundance of Myxococcales was 10^7^ copies per gram soil in rhizosphere of the greenhouse mesocosm experiment, refreshing the understanding of myxobacterial abundance by the culture-dependent method. Furthermore, with the increase of myxobacterial sequences, specific primers would be expected and contribute to the precise quantification of the myxobactrial abundance.

Most of the myxobacterial sequences from subtropical and tropical forest soil in this study were uncultured. Our results showed that the abundance of Sorangiineae and Nannocystineae were much higher than that of Cystobacterineae, which is consistent with the results indicated by prokaryotic 16S rRNA transcripts [48]. The low abundance of Cystobacterineae illustrated by culture-independent methods indicates the biases of the culture-dependent method, because species from Cystobacterineae are frequently isolated [49]. We detected sequences from all three known suborders of Myxococcales based on soil 16S rDNA sequencing in this study, but 17%−43% Myxococcales sequences were unclassified myxobacterial ASVs which could not be assigned to the three known suborders. However, the retrieved myxobacterial sequences from 16S rDNA library are strongly dependent on sequencing depth. We suggest the combination of specific primer sequencing [50] and 16S rDNA sequencing method to better understand the myxobacterial community in the future study. These results imply the necessity of more efforts on the less characterized myxobacterial taxa via culture-dependent and -independent methods.

### 4.2. Effect of Soil Bacteria on Myxobacterial Community

Microorganisms are involved in complex interactions with other organisms and their habitat. In addition to the frequently reported environmental factors, recent study has shown that microbial interactions, such as auxotrophies and nutrient requirements among members of the microbial community, are a biotic force in shaping the microbial community [26]. It is expected that the soil myxobacteria community may be regulated by prey, because of their deficiency in the biosynthesis of riboflavin and branched-chain amino acids [51]. The strict secondary labelling of soil myxobacteria from plant-derived carbon indicated by rRNA-SIP implied their feeding on labeled bacterial biomass [52]. We found the enriched myxobacteria with increased bacterial 16S rRNA gene copy numbers due to the plant presence in the greenhouse experiment. Considering the predation and the dependence on macromolecular of myxobacteria, it seems that the plants indirectly affect myxobacteria abundance via rhizosphere bacteria, and the increased abundance of myxobacteria was attributed to the higher number of prey bacterial cells. In accordance with our results, the abundance of bacterivores (with Myxococcales as dominant) was positively associated with abundance of prey bacteria during beech litter decomposition [48].

We found a non-negligible explanation of bacterial community composition on myxobacterial community. The significant associations between bacteria and myxobacteria indicated links among bacteria taxa at the community level. A previous study reported that bacteria taxa was the biotic factor in shaping the predator *Bdellovibrio* community, a parasitic predator group phylogenetically related to myxobacteria within Deltaproteobacteria, and it was demonstrated that prey cells with big size may contribute to the selection of predators [23]. However, prey cell size may not be the reason for the bacterial regulation of myxobacteria, because of their “wolf pack hunting” strategy [1,2]. The preferential predation of micropredators on prey may explain the bacteria as factors affecting the predatory microbial community. The significant correlations of myxobacterial abundance and community composition with bacteria in forest soil observed in this study, while not establishing a cause and effect relationship, revealed the direct correlative associations between the potential preys and predators at the community level. Indeed, the different bacterial prey species can be consumed by different myxobacteria, with the prey bacteria *Pseudomonas putida* supporting much more diverse myxobacteria than *Arthrobacter globiformis* [53]. These results indicated that soil bacteria could influence myxobacterial community via microbial food web. However, further confirmation study is needed to distinguish the biological predation from scavenging.

The bacterial co-occurrence network was used as a tentative exploration of the pairwise interactions between myxobacteria and other bacteria in this study. We are aware that interpreting network interactions from a biological or ecological perspective is difficult, and the potentially predatory interactions need further confirmation. Still, we tried to link the interactions from the network with biological principles to explore the potential effect of interactions on the myxobacterial community. The network in this study indicated strong links between myxobacteria and bacteria belonging to taxa from Alphaproteobacteria, Betaproteobacteria, Gemmatimonadetes, Acidobacteria and other Gram-negative groups in forest soil, which may imply the potentially preferential preys. Morgan et al. [20] reported that *Myxococcus* from the Cystobacterineae suborder were supported more by Gram-negative prey species than by Gram-positive species. We also detected all the Cystobacterineae nodes linked with Gram-negative species within the network. However, the general conclusion that Cystobacterineae could be supported more efficiently by Gram-negative preys still needs more evidence. It is not known whether myxobacteria from Nannocystineae suborder can prey on bacteria, but these taxa are known for agar degradation [54]. Haliangiaceae from Nannocystineae was shown to be efficiently supported by *Arthrobacter globiformis*, a Gram-positive actinobacteria [53]. We also detected a link between taxa from Haliangiaceae and the Gram-positive bacteria from Ktedonobacteria, the members of which have an actinomycetes-like morphology with branched mycelia and spores [55]. The links with Gram-positive actinomycetes or actinomycetes-like bacteria indicated that these preys may adopt a different regulation manner affecting Haliangiaceae because they are both antimicrobial compound producers, which may need further study.

### 4.3. Effect of Soil Abiotic Factors on Myxobacterial Community

Climate factors and soil characteristics have been reported to correlate with the abundance of myxobacteria [16]. We also found correlations between abiotic factors and myxobacterial community in this study. The proportion of variance explained by the measured soil properties, albeit as a minor effect compared with bacterial factor, showed their direct associations with soil myxobacterial communities. The minor effect of abiotic factors could be expected due to the similar soil type and climate conditions of the sampling sites in this study. It is probable that the effect size of soil abiotic factors may be greater in more distinct systems. The high adaptability of myxobacteria and their dependence on prey bacteria also weaken the influence of abiotic factors [5].

In this study, nearly 80% of variation in the subtropical and tropical myxobacterial community could not be explained by the measured abiotic and bacterial factors. We can find some reports about the bacteria community or bacterial taxa with comparable explained variances to our result [56,57,58,59]. On the one hand, some soil variables that were not measured in this study may be influential. However, soil nutrient content showed a small variance among our sampling sites, which may exert a less important influence than pH and SOM. We also assessed the effects of other soil variables in the greenhouse mesocosm experiment and confirmed the predominant effect of pH and SOM. On the other hand, stochastic processes (such as immigration, mutations, and extinction) also contribute to variation in the bacterial community [60], which may also influence the assembly of myxobacterial population. In addition, identification of metabolic activity and dormant myxospores of myxobacteria based on DNA were not available. Further studies based on metabolism may improve the understanding of myxobacterial ecology.

## 5. Conclusions

Microorganisms interact with the environment and other organisms, shaping biodiversity. Little is known about the relative contributions of biotic and abiotic factors in microbial assembly. As one of the most abundant micropredators, myxobacteria show intricate associations with prey, making myxobacteria an optimal group to study bacterial interactions at the community level. In this study, we characterized the relative abundance, community composition of myxobacteria, and the myxobacterial correlations with bacteria and soil properties (pH and SOM) in subtropical and tropical forest soil from South China. Our results showed that both soil samples from natural forests and rhizosphere soil from a greenhouse mesocosm experiment revealed a slightly stronger effect of bacterial community on myxobacteria than that of soil chemical properties. The co-occurrence of myxobacteria and other bacteria within the nonrandom network indicated that the myxobacteria preferential predation on different bacteria may explain how bacterial community composition affect myxobacterial community, which still need further biological confirmation. As an important component in soil carbon flow, predatory myxobacteria community can be regulated via micro-food web, providing new insight into myxobacterial ecology.

## Figures and Tables

**Table 1:** Correlations between myxobacteria and soil bacterial groups at the phylum level of subtropical and tropical forest soil. The relationship between soil bacteria and myxobacterial abundance was indicated by Spearman coefficient, and the correlation between bacterial abundance and myxobacterial community (pairwise Bray-Curtis distance) was indicated by Spearman correlation using the Mantel test.

	Myxobacterial Abundance		Myxobacterial Community Composition	
	r	*p*	r	*p*
Proteobacteria	−0.021	0.865	0.297	<0.001
Acidobacteria	−0.495	<0.001	0.216	<0.001
Actinobacteria	−0.112	0.351	0.169	<0.001
Bacteroidetes	0.316	0.007	0.185	<0.001
Chloroflexi	0.255	0.032	0.242	<0.001
Firmicutes	0.415	<0.001	0.156	<0.001
Gemmatimonadetes	0.694	<0.001	0.106	0.005
Verrucomicrobia	−0.063	0.602	0.05	0.121

## Supplementary Materials

The following are available online at https://www.mdpi.com/2076-2607/8/9/1387/s1, Table S1: The scores and variance percentages of axis by principal coordinate analysis (PCoA) of bacterial community from forest soil in the field investigation. Bact1 and Bact2 represent the first two axis were used as indicators of bacterial community. Table S2: The scores and variance percentages of the axis indicated by principal coordinate analysis (PCoA) of soil bacterial community from rhizosphere in the greenhouse mesocosm. Bact1 and Bact2 represent the first two axes used as indicators of bacterial community. Table S3: Correlation between ordination axis and constrained factors in the greenhouse mesocosm. Monte Carlo permutation test was used to evaluate significance. R^2^ means the effect size of specific factor on soil myxobacteria, and *p* < 0.05 mean significant factor effect on myxobacterial community. Bact1 and Bact2 indicate the first and second axes generated from principal coordinate analysis (PCoA) using soil microbial community composition data. Bact_abundance mean the copy numbers of soil bacteria from qPCR. Table S4: Topological properties of the plant-associated co-occurrence network of bacterial community and the random network from rhizosphere in the greenhouse mesocosm. Figure S1: Redundancy analysis (RDA) of soil myxobacteria community composition constrained by soil bacterial community and soil property in the greenhouse mesocosm. Bact1 and Bact2 mean the first and second principal components generated from principal coordinate analysis (PCoA) using soil microbial community composition data. Bact_abundance mean the copy numbers of soil bacteria from qPCR.
