# Supplementary material for: Both Soil Bacteria and Soil Chemical Property Affected the Micropredator Myxobacterial Community: Evidence from Natural Forest Soil and Greenhouse Rhizosphere Soil
Source: Microorganisms. 2020 Sep 10;8(9):1387. doi: 10.3390/microorganisms8091387 (PMC7563646; doi:10.3390/microorganisms8091387)
Supplement: Supplementary file 1 [file microorganisms-08-01387-s001.zip › Supplementary Materials.docx]

**Table S1** The scores and variance percentages of axis by principal coordinate analysis (PCoA) of bacterial community from forest soil in the field investigation. Bact1 and Bact2 represent the first two axis were used as indicators of bacterial community.

|  | **Bact1** | **Bact2** |
| --- | --- | --- |
| CB1 | 0.087 | -0.096 |
| CB10 | 0.095 | -0.078 |
| CB2 | 0.091 | -0.108 |
| CB21 | 0.09 | -0.059 |
| CB23 | 0.092 | -0.100 |
| CB24 | 0.078 | -0.069 |
| CB25 | 0.082 | -0.101 |
| CB26 | 0.086 | -0.130 |
| CB27 | 0.087 | -0.084 |
| CB28 | 0.076 | -0.096 |
| CB3 | 0.095 | -0.125 |
| CB31 | 0.074 | -0.049 |
| CB35 | 0.099 | -0.111 |
| CB36 | 0.100 | -0.094 |
| CB37 | 0.063 | -0.081 |
| CB4 | 0.088 | -0.099 |
| CB42 | 0.043 | -0.121 |
| CB5 | 0.090 | -0.112 |
| CB6 | 0.090 | -0.135 |
| CB7 | 0.080 | -0.124 |
| CB8 | 0.078 | -0.121 |
| CB9 | 0.094 | -0.118 |
| DL1 | 0.036 | 0.018 |
| DL10 | 0.013 | 0.083 |
| DL11 | 0.037 | 0.102 |
| DL12 | 0.043 | 0.047 |
| DL13 | 0.025 | 0.086 |
| DL14 | 0.029 | 0.052 |
| DL15 | 0.028 | 0.093 |
| DL16 | 0.019 | 0.034 |
| DL17 | 0.030 | 0.022 |
| DL18 | 0.014 | 0.053 |
| DL3 | -0.132 | 0.094 |
| DL4 | 0.032 | 0.085 |
| DL5 | 0.043 | 0.020 |
| DL6 | 0.032 | 0.067 |
| DL8 | 0.043 | 0.130 |
| DL9 | 0.029 | 0.016 |
| ES1 | 0.086 | 0.338 |
| ES10 | 0.059 | 0.085 |
| ES2 | 0.074 | 0.293 |
| ES3 | 0.076 | 0.208 |
| ES5 | 0.099 | 0.514 |
| ES7 | 0.058 | 0.106 |
| ES8 | 0.059 | 0.092 |
| LM10 | -0.102 | 0.069 |
| LM11 | -0.155 | 0.007 |
| LM12 | -0.096 | 0.000 |
| LM13 | -0.058 | 0.055 |
| LM14 | -0.025 | 0.074 |
| LM16 | -0.041 | 0.064 |
| LM2 | 0.035 | -0.101 |
| LM20 | -0.109 | -0.101 |
| LM21 | 0.021 | -0.066 |
| LM22 | -0.050 | -0.069 |
| LM23 | 0.027 | -0.060 |
| LM24 | 0.030 | -0.102 |
| LM25 | 0.045 | -0.027 |
| LM26 | -0.160 | -0.115 |
| LM27 | 0.022 | -0.104 |
| LM28 | -0.085 | -0.078 |
| LM29 | -0.099 | -0.120 |
| LM3 | -0.274 | 0.045 |
| LM30 | -0.187 | 0.106 |
| LM31 | -0.379 | 0.046 |
| LM32 | -0.004 | 0.083 |
| LM4 | -0.002 | 0.052 |
| LM5 | -0.020 | 0.084 |
| LM7 | -0.156 | -0.013 |
| LM8 | -0.442 | -0.100 |
| LM9 | -0.427 | -0.051 |
| Explained variance (%) | 30.91 | 26.13 |

**Table S2** The scores and variance percentages of the axis indicated by principal coordinate analysis (PCoA) of soil bacterial community from rhizosphere in the greenhouse mesocosm. Bact1 and Bact2 represent the first two axis were used as indicators of bacterial community.

|  | **Bact1** | **Bact2** |
| --- | --- | --- |
| PN1 | 0.102 | 0.348 |
| PN2 | 0.161 | 0.306 |
| PN3 | 0.198 | 0.184 |
| FA1 | -0.194 | -0.191 |
| FA2 | 0.148 | 0.374 |
| FA3 | -0.017 | 0.099 |
| LP1 | 0.163 | -0.005 |
| LP2 | 0.185 | -0.035 |
| LP3 | 0.168 | 0.060 |
| SG1 | -0.260 | 0.057 |
| SG2 | -0.264 | -0.057 |
| TP1 | -0.300 | -0.182 |
| TP2 | -0.216 | 0.094 |
| TP3 | -0.312 | -0.294 |
| MS1 | -0.181 | 0.041 |
| MS2 | -0.152 | 0.092 |
| MS3 | -0.173 | 0.122 |
| CK1 | 0.321 | -0.166 |
| CK2 | 0.417 | -0.334 |
| CK3 | 0.206 | -0.512 |
| Explained variation (%) | 39.72 | 17.13 |

**Table S3** Correlation between ordination axis and constrained factors in the greenhouse mesocosm. Monte Carlo permutation test was used to evaluate significance. R^2^ means the effect size of specific factor on soil myxobacteria, and P < 0.05 mean significant factor effect on myxobacterial community. Bact1 and Bact2 mean the first and second axis generated from principal coordinate analysis (PCoA) using soil microbial community composition data. Bact_abundance mean the copy numbers of soil bacteria from qPCR.

|  | RDA1 | RDA2 | R^2^ | P |
| --- | --- | --- | --- | --- |
| Bact1 | 0.966 | -0.259 | 0.795 | <0.001 |
| Bact_abundance | 0.663 | 0.749 | 0.529 | 0.001 |
| Bact2 | 0.693 | 0.721 | 0.476 | 0.003 |
| SOM | 0.177 | 0.984 | 0.449 | 0.005 |
| pH | 0.998 | -0.054 | 0.151 | 0.245 |

**Table S4** Topological properties of the plant-associated co-occurrence network of bacterial community and the random network from rhizosphere in the greenhouse mesocosm.

|  | **Empirical network** | **Random network** |
| --- | --- | --- |
| Total nodes | 322 | -- |
| Total links | 463 | -- |
| R^2^ of power-law | 0.856 | -- |
| Average clustering coefficient | 0.164 | 0.019 ± 0.005 |
| Average path distance | 6.280 | 4.367 ± 0.075 |
| Harmonic geodesic distance | 4.798 | 3.863 ± 0.053 |
| Modularity | 0.739 | 0.617 ± 0.008 |


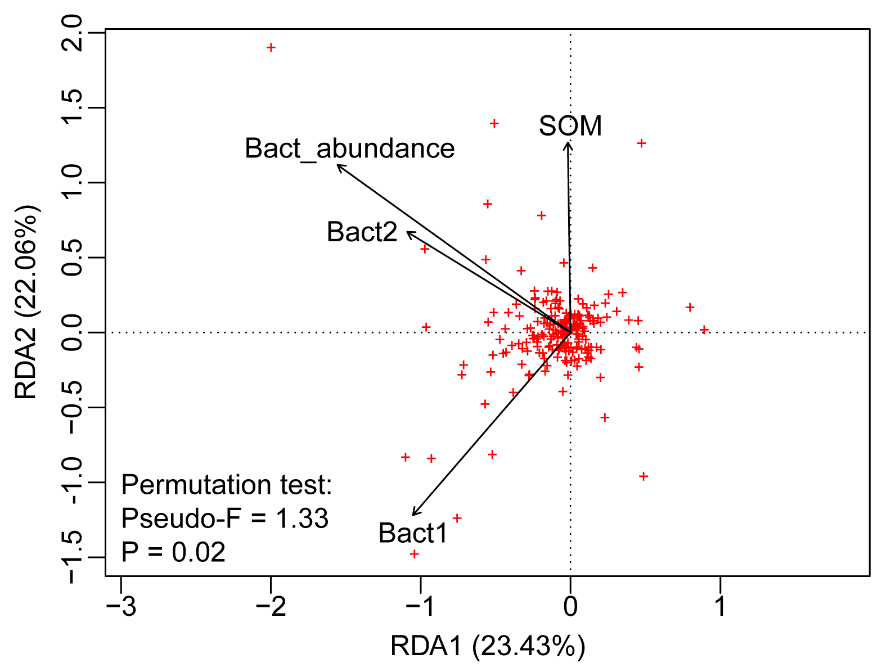


**Figure S1** Redundancy analysis (RDA) of soil myxobacteria community composition constrained by soil bacterial community and soil property in the greenhouse mesocosm. Bact1 and Bact2 mean the first and second principal components generated from principal coordinate analysis (PCoA) using soil microbial community composition data. Bact_abundance mean the copy numbers of soil bacteria from qPCR.
